# Gramian Angular Field and Convolutional Neural Networks for Real-Time Multiband Spectrum Sensing in Cognitive Radio Networks

**DOI:** 10.3390/s25123580

**Published:** 2025-06-06

**Authors:** Yanqueleth Molina-Tenorio, Alfonso Prieto-Guerrero, Enrique Rodriguez-Colina, Luis Alberto Vásquez-Toledo, Omar Alejandro Olvera-Guerrero

**Affiliations:** 1 Electrical Engineering Department, Universidad Autónoma Metropolitana-Iztapalapa, Av. Ferrocarril San Rafael Atlixco 186, Mexico City 09310, Mexico; yanqueleth@xanum.uam.mx (Y.M.-T.); erod@xanum.uam.mx (E.R.-C.); lvasquezt@xanum.uam.mx (L.A.V.-T.); 2 Universidad Politécnica de Chiapas, Carretera Tuxtla Gutierrez-Portillo Zaragoza km 21+500, Suchiapa 29150, Mexico; oolvera@ib.upchiapas.edu.mx (O.A.O.-G.)

**Keywords:** cognitive radio networks, multiband spectrum sensing, gramian angular field, convolutional neural networks

## Abstract

Multiband spectrum sensing in a cooperative environment is a novel solution for efficient spectrum resource management under the cognitive radio networks (CRNs) paradigm. This paper presents a distinctive framework where a central entity collects power spectral density data from multiple geographically distributed secondary users and applies the Gramian angular field (GAF) summation method to transform the time-series data into image representations. A major contribution of this work is the integration of these GAF images with a convolutional neural network (CNN), enabling precise and real-time detection of primary user activity and spectrum occupancy. The proposed approach achieves 99.6% accuracy in determining spectrum occupancy, significantly outperforming traditional sensing techniques. The main contributions of this study are (i) the introduction of GAF-based image representations for cooperative spectrum sensing in CRNs; (ii) the development of a CNN-based classification framework for enhanced spectrum occupancy detection; and (iii) the demonstration of superior detection performance in dynamic, real-time environments.

## 1. Introduction

Several studies have shown that a large part of the radio spectrum remains underutilized, depending on time and location. This problem led to the introduction of the cognitive radio (CR) paradigm two decades ago [1]. However, this idea remains relevant today due to the exponential growth of wireless communication devices that has generated an ever-increasing demand for spectral resources. CRs are radio devices (known as secondary users; SUs) that dynamically adapt to the spectral environment, identifying free frequency bands and using them without causing interference to licensed users (also known as primary users; PUs). The cognitive radio paradigm is a promising solution to optimize spectrum use, improving spectral efficiency and facilitating the coexistence of multiple communication systems.

Fundamental for the correct operation of a cognitive radio, in order to detect primary users, is spectrum monitoring or sensing. This process involves the detection and analysis of signals present in different frequency bands to determine their availability. Monitoring can be performed considering a single band, containing a single primary user, or a multiband, where many primary users could be present. This latter situation is more complex but also more efficient as it provides a wide point of view for spectrum use. Multiband monitoring allows for not only the identification of the presence of primary users, but also the determination of occupancy patterns in time, frequency, and space, which is vital in dynamic environments for CRNs.

Various approaches have been explored to address multiband spectrum sensing. Traditional methods are based on energy detection techniques, covariance-based detection, or feature detection [2,3,4,5]. Although these methodologies have proven to be effective under certain scenarios, they have limitations when faced with low signal-to-noise ratio (SNR) environments or when they need to simultaneously analyze large frequency bands. To overcome these limitations, approaches based on mixtures of classical digital signal processing (DSP) and machine learning (ML) techniques have been introduced, which allow complex patterns to be extracted from the spectrum, improving detection capacity. In particular, CNNs have shown promising results in signal classification and spectral occupancy identification, taking advantage of their ability to extract spatial and temporal features from data [6]. Recent works, such as [7,8], emphasize how deep learning techniques significantly enhance multiband spectrum sensing under dynamic and noisy conditions. This approach requires advanced systems capable of processing large volumes of spectral information in real time, thus posing new challenges in terms of computational efficiency and detection accuracy.

In this context, methods such as power spectral density (PSD)-based frequency- domain sensing have been proposed, which outperforms conventional energy sensing methods by identifying primary users under low-SNR conditions [9]. To address secondary user hardware limitations, random and adaptive sensing strategies have been developed, with the latter leveraging PU traffic patterns to improve sensing performance [10]. These strategies allow devices with limited capabilities to efficiently sample the spectrum, maximizing the probability of detection.

The use of software-defined radio (SDR) platforms has also enabled the flexible implementation of advanced spectral analysis techniques, facilitating real-time signal identification. These programable systems can dynamically adjust their monitoring parameters, responding favorably to changing environments. In this regard, approaches based on deep neural networks (DNNs) have been explored to improve signal classification accuracy, allowing for the identification of nonlinear and dynamic patterns in the spectrum [11]. Among the most notable techniques are CNNs and recurrent neural networks (RNNs). For example, the combination of a CNN for feature extraction and long short-term memory (LSTM) for temporal correlation analysis has been shown to improve accuracy and robustness in cooperative spectrum sensing in vehicular networks, especially in low-SNR environments [12]. Furthermore, a CNN-based approach has been proposed incorporating information from the covariance matrix, leveraging hidden correlations between sub-bands for more accurate detection, even in the presence of noise uncertainty [13]. In addition, a recent multi-user collaborative spectrum sensing model based on a CNN-LSTM hybrid architecture, enhanced with a multi-head self-attention mechanism, has demonstrated superior sensing accuracy and efficiency across dynamic environments [14]. This model not only leverages CNN’s feature extraction and LSTM’s handling of sequential data, but also optimizes information flow among users, reducing sensing error rates significantly compared to other deep learning methods, particularly under low-power conditions. Additionally, recent works have explored optimizing cognitive radio networks not only for detection accuracy but also for timely information delivery, such as minimizing the Age of Information in ambient backscatter-assisted energy-harvesting CRNs using deep neural networks and advanced reinforcement learning strategies [15].

Furthermore, references [16,17] provide comprehensive reviews and propose novel RIS-enhanced architectures and deep learning frameworks, highlighting new opportunities and challenges for next-generation CRNs. Together, these advancements, along with the flexibility of SDR platforms to cover multiple frequency bands simultaneously, have made a significant difference in spectrum monitoring, overcoming the limitations of traditional methods and enabling more accurate and efficient detection in complex environments. Despite these advancements, there is still a critical need for lightweight and efficient methods that can operate under low-SNR conditions and process high-dimensional spectral data in real time, especially when leveraging centralized architectures with multiple SDRs. This motivates the development of novel frameworks that combine efficient feature extraction and robust learning models, aiming to enhance the detection of primary users while minimizing computational burden. Based on these facts, this work proposes a novel centralized multiband monitoring network that integrates advanced digital signal processing and CNNs to determine radio spectrum occupancy using SDR technology in real-time scenarios. The key novelty of this work lies in the innovative use of the Gramian angular field (GAF) transformation, which converts one-dimensional spectral sequences into colorized images, enabling efficient feature extraction and robust classification. In addition, it has been demonstrated that with a simple CNN architecture it can match or outperform more commonly used complex models, offering an efficient solution for one-dimensional sequence analysis [18,19,20,21]. In this way, in this work a GAF-CNN-based detection algorithm is proposed in order to improve PU signal detection in low-SNR environments by extracting and preprocessing spectrum (one-dimensional sequence) features. The combination of GAFs and a CNN provides a robust system, capable of identifying complex patterns and adapting to varying spectrum conditions (real-time situations). Furthermore, the centralized architecture facilitates the integration of information from multiple sensors (SDRs), forming a cognitive radio network, allowing for more accurate and efficient spectrum monitoring in a specific geographic area. The results show that the use of GAFs combined with a CNN significantly improves spectral occupancy detection in comparison with conventional methods. This research focuses on evaluating the performance of the proposed system in terms of detection accuracy and generalization ability in different spectral environments. In this way, this approach represents a significant step towards the implementation of smarter and more adaptive cognitive radio systems, capable of responding to the growing demands of wireless communication systems. Recent studies using ensemble classifiers and feature-assisted sensing further confirm the relevance of combining machine learning and advanced sensing strategies to improve cognitive radio performance [22,23].

This work is structured as follows: The first section of the paper is the Introduction, where the topic and aims of this work are described. Section 2 addresses the fundamental bases that support the development of the proposed methodology. Section 3 presents the base methodology, developed previously by the authors, as a contextual reference. Section 4 presents the development of the idea (methodology) that constitutes the core of this study. In Section 5, the real-time scenario and the experimental results are presented. Finally, conclusions are delivered in Section 6.

## 2. Theoretical Background

In this section, the theoretical bases used for the development of the methodology for cooperative multiband spectrum sensing are succinctly introduced.

### 2.1. Gramian Angular Field

Gramian angular field is a technique used to transform a one-dimensional sequence into two-dimensional one (an image) [24], representing some kind of correlation between each pair of values from the one-dimensional sequence, in order to explore patterns in the data. This technique is especially useful in machine learning tasks such as CNNs, that normally work with models constructed with images instead of time series (or one- dimensional sequences, in general). A GAF is constructed as follows:

Consider *N* observations from a real-valued one-dimensional signal $X=\left\{x_{1},x_{2},\ldots ,x_{N}\right\}$.

The samples of *X* are scaled in the sequence $\tilde{X}$ so that all values fall in the interval [0, 1] or [1, −1] as follows:

(1) $$\begin{array}{l} {\tilde{x}}_{i}=\frac{\left(x_{i}-\max\left(X\right)\right)+\left(x_{i}-\min\left(X\right)\right)}{\max\left(X\right)-\min\left(X\right)}\;\in \left[1,-1\right]\\ \mathrm{or}\\ {\tilde{x}}_{i}=\frac{x_{i}-\min\left(X\right)}{\max\left(X\right)-\min\left(X\right)}\;\in \left[0,1\right] \end{array}$$

In this way, it is possible to represent the rescaled sequence $\tilde{X}$ in polar coordinates by encoding each value of $\tilde{X}$ as the angular cosine and the discrete sample number as the radius, as follows:

(2) $$\begin{array}{l} \theta_{i}=\arccos\left({\tilde{x}}_{i}\right),\;{\tilde{x}}_{i}\in \tilde{X}\\ r_{i}=\frac{i}{N} \end{array}$$

where *i* is the discrete time sample, *N* is the sequence length, $\theta_{i}$ denotes the arccosine, and $r_{i}$ is the radius of the *i*-th rescaled value of the original sequence.

Given the rescaled sequence in the polar coordinate system, the angular perspective is exported considering the trigonometric sum/difference between each point to identify the temporal correlation within different discrete intervals, thus giving rise to the Gramian angular summation field (*GASF*) and the Gramian angular difference field (*GADF*) as follows:

(3) $$\begin{array}{c} GASF=\left[\cos\left(\theta_{i}+\theta_{j}\right)\right]={\tilde{X}}^{T}\cdot \tilde{X}-{\sqrt{I-{\tilde{X}}^{2}}}^{T}\cdot \sqrt{I-{\tilde{X}}^{2}}\\ GADF=\left[\cos\left(\theta_{i}-\theta_{j}\right)\right]={\sqrt{I-{\tilde{X}}^{2}}}^{T}\cdot \tilde{X}-{\tilde{X}}^{T}\cdot \sqrt{I-{\tilde{X}}^{2}} \end{array}$$

where *T* indicates transpose, and *I* is the unit row vector [1 1 1…1].

**Figure 1:**
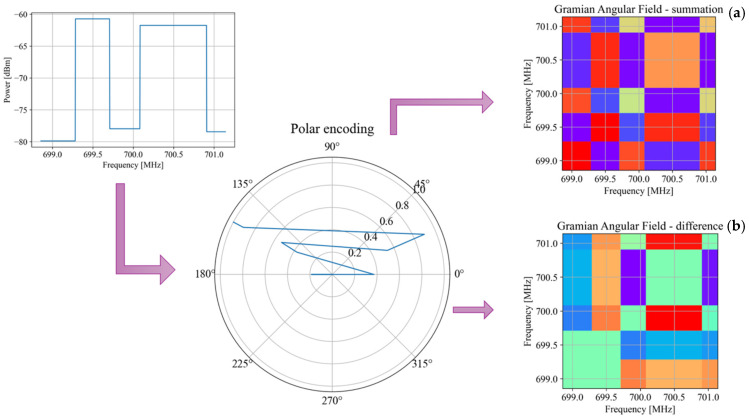
GAF in its (**a**) summation version and (**b**) difference version when applied to a discrete signal containing power averages for different frequency bands.

Writing the summation or difference of different angles in Equation (3) as inner products, allows us to clearly observe the correlation of two vectors. In this way, the *GASF* and *GADF* matrices reflect the correlation between the angle sum and angle difference, respectively. The GAF is a matrix representation that encodes a discrete set of points in a square image. This image captures the relationships and dependencies between the points in the set, making it easier to analyze them considering image processing methods. Figure 1 shows the result of applying the GAF in its different modes (sum and difference) to a discrete signal representing power averages in several frequency intervals (bands).

### 2.2. Convolutional Neural Networks

In recent years, CNNs [25] have emerged as a key tool in deep learning, especially for image processing, allowing for the efficient identification of relevant patterns and features, for example, in object recognition, image segmentation, and visual analysis tasks [26,27].

**Figure 2:**
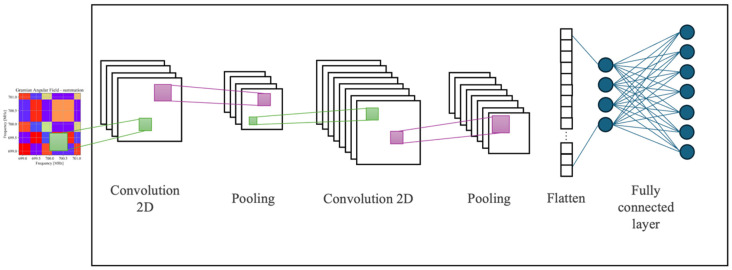
Structure of a convolutional neural network, in which its different layers can be distinguished. In this example, the input for this typical CNN structure is a *GASF* matrix.

A typical CNN consists of several layers (see Figure 2): the convolutional layer, which extracts local features from the image using filters; and the pooling layer, which reduces the spatial dimensions of the feature map, preserving essential information. After several convolutional and pooling layers, the features are flattened into a dense vector, which is passed to the fully connected layers to perform classification. Finally, the network adjusts the weights using backpropagation and optimization algorithms, such as Stochastic Gradient Descent (SGD), to improve the accuracy of estimates [6]. The activation function (such as ReLU, sigmoid, or tanh), applied in every layer, introduces nonlinearity and improves learning by avoiding the vanishing gradient problem.

In this work, the use of a CNN for processing matrices resulting from applying the *GASF* method for spectrum occupation estimation is proposed.

## 3. Previous Work

A previous methodology [28] to detect PUs in a cooperative CRN was developed and implemented in a real-time scenario by the authors. This CRN consists of a central entity coordinating several interconnected SDR devices, working as SUs, capable of sensing a wide range of frequencies in a specific geographic region. This previous methodology, briefly explained in the following paragraphs, serves as a basis and is modified with the introduction of the GAF method and the estimation of spectral occupation with a CNN.

**Figure 3:**
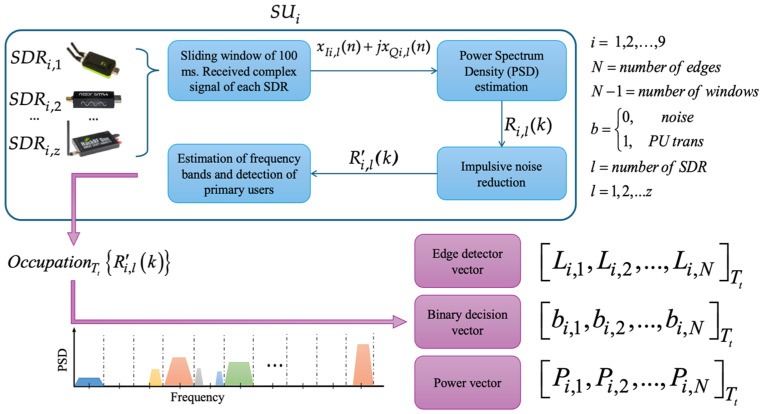
Operation of each secondary user in the implemented CRN.

In Figure 3 it is shown how each secondary entity (SU), equipped with heterogeneous technologies (SDRs), performs multiband spectrum sensing (MBSS), obtaining three fundamental vectors: (i) the edge detector vector ${\left[L_{i,1},L_{i,2},\ldots ,L_{i,N}\right]}_{T_{t}}$, which stores the edge frequencies at which the presence of PUs can be identified over (*N* − 1) dynamically detected windows; (ii) the binary decision vector ${\left[b_{i,1},b_{i,2},\ldots ,b_{i,N-1}\right]}_{T_{t}}$, which contains the binary decisions corresponding to delimited bands, which indicate the detection of noise or the possible transmission of PUs; and (iii) the power vector ${\left[P_{i,1},P_{i,2},\ldots ,P_{i,N-1}\right]}_{T_{t}}$, which records the average received power for each classified window, i.e., for each binary decision. Thus, the binary and power decision vectors are of an equivalent size. In addition to the task of monitoring the radio spectrum and analyzing the behavior of PUs in a specific geographic region, this cooperative CR network aims to mitigate the hidden terminal problem, thus ensuring a more efficient and reliable use of the spectrum.

**Figure 4:**
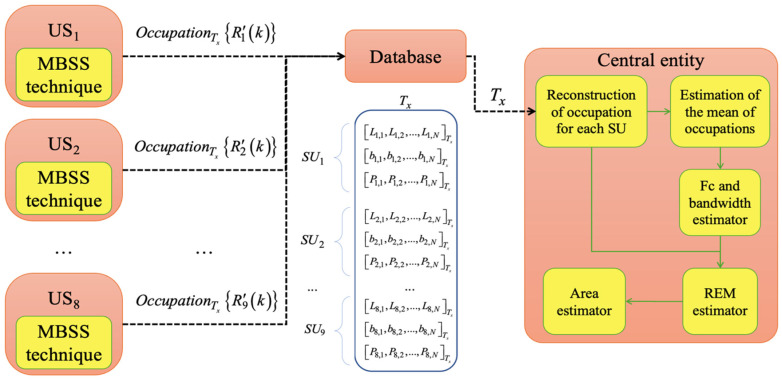
General scheme of the implemented CRN.

The complete CRN integrates three main components (see Figure 4): first, a central entity, collecting all the locally processed sensed data (i.e., spectrum occupancy, frequency band edges, and estimated power vectors) from each SU. Second, a database, systematically storing the received information at specific time intervals for further analysis. Finally, data processing, realized by the central entity, employing advanced computational techniques to analyze spectrum utilization (occupation) and optimize resource allocation, in order to determine the geographic regions occupied by PUs in the radio spectrum through the construction of radio environment maps (REMs).

## 4. Proposed Methodology

**Figure 5:**
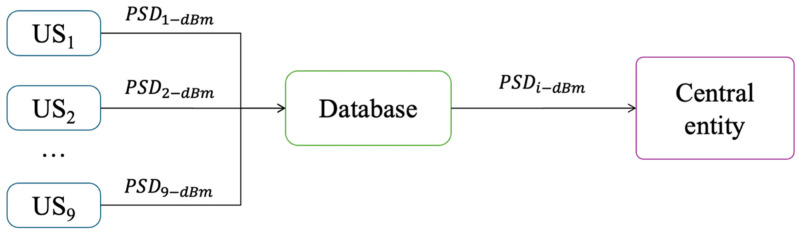
Sharing to the central entity of estimated average PSDs from data sensed by each SU.

In this proposal, the previous methodology (Section 3) is modified, integrating the GAF method and a CNN to enhance the system’s performance in detecting PUs. This new proposal considers first that each SU shares only the average PSD, resulting from the estimated PSD from each analyzed dynamically sized window, with the central entity obtained through the database, as shown in Figure 5.

**Figure 6:**
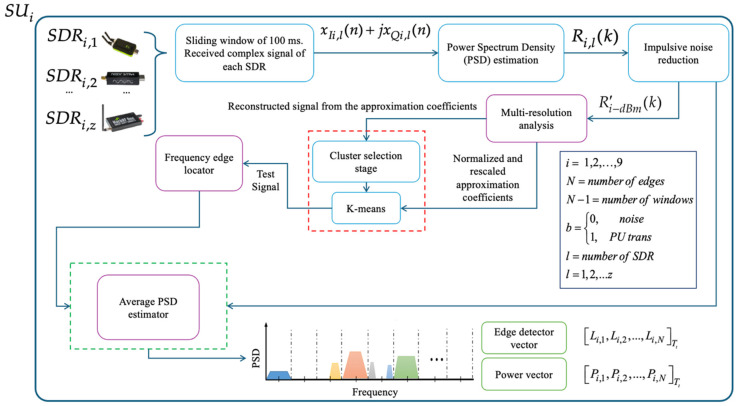
Flowchart of the operation of the *i*-th SU of the proposed CRN.

This means that the secondary entities perform less processing, in comparison with the previous methodology, because they do not determine the occupation of the analyzed spectrum. The complete modified process realized by each SU, forming the CRN, is indicated in Figure 6 and described by Algorithm 1. Figure 6 summarizes the internal processing steps performed by each SU, highlighting how the combination of multiresolution analysis (MRA) and clustering enables a more adaptive and dynamic segmentation of the spectrum. This figure illustrates not only the processing flow, but also emphasizes the reduced computational burden on each SU compared to previous approaches, as occupation detection is centralized.
**Algorithm 1.** Operation of the *i*-th SUStep 1.1. Given the PSD in a dBm scale $R_{i-dBm}^{\prime }\left(k\right)$, an MRA is performed over it. In this way, the approximation coefficients at a certain decomposition level and the detail coefficients at different decomposition levels are obtained. Furthermore, the signal $R_{i-dBm}^{\prime }\left(k\right)$ is reconstructed using only the approximation coefficients, thus providing the signal trend. These approximation coefficients are also scaled and normalized for further processing.Step 1.2. The reconstructed PSD with the MRA, the scaled and normalized approximation coefficients obtained in the previous step, in addition to a cluster selection stage and the K-means algorithm, allow the construction of the test signal. This signal, varying in a binary way, clearly shows state changes occurring in the original PSD.Step 1.3. Next, the test signal is used to identify the points where a state change occurred. These state changes, representing singularities in the signal, conform to dynamically sized windows (segments of the test signal) for the analysis.Step 1.4. Since the dynamic windows define frequency boundaries, the mean value of the PSD within each window is computed, forming the average PSD signal. Step 1.5. Finally, the information is shared with the central entity via the database. The shared data include the following: The edge detection vector, which indicates the exact points where a change in the signal occurred. The power vector, which represents the average PSD value within each dynamic window defined by the frequency limits. These vectors are stored and managed in a centralized database, which facilitates their access for subsequent analysis and decision making in the spectrum detection system.

The sliding window, PSD estimator, and impulsive noise reduction modules are responsible for configuring the SDRs to allow the collection of samples, estimate the PSD via the Welch method, and alleviate the problem of the impulsive noise [29] introduced by the SDRs’ hardware, respectively.

**Figure 7:**
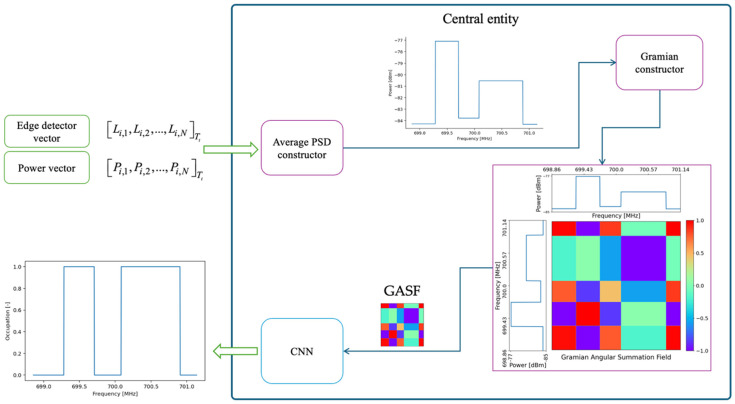
Operating scheme of the central entity.

The central entity processes the edge and power vectors, as illustrated in Figure 7, by applying the following Algorithm 2.
**Algorithm 2.** Central Entity ProcessesStep 2.1. Average PSD reconstruction: From the vectors extracted in Algorithm 1, the average PSD, formally denoted as $PSD\text{\_}mean\left(k\right)$, is reconstructed. Where k represents the frequency index in the spectral domain.Step 2.2. Signal transformation into a two-dimensional representation: The discrete signal $PSD\text{\_}mean\left(k\right)$ is subjected to a transformation using the GAF method, specifically in its summation variant, generating the *GASF* matrix. This matrix preserves the spectral information of the signal, allowing the following to be captured:Correlation structures between the samples of the discrete series.Dynamic evolution of processed signal behavior.Facilitation of pattern interpretation using image analysis techniques.Step 2.3. Spectrum occupancy inference using a CNN: The *GASF* matrix is fed into a CNN, in order to extract spatial and spectral features relevant for spectral occupancy classification. The output of the model is a discrete binary signal of equal length to $PSD\text{\_}mean\left(k\right)$, where each value indicates the spectral occupancy at a given frequency:1: Indicates that the spectrum is occupied at the corresponding frequency.0: Indicates that the spectrum is free at that frequency.

The main strength of the proposed method lies in its hybrid approach, combining lightweight processing at the SUs (by only sharing averaged PSDs) with a powerful central analysis using the GAF and CNN. This significantly reduces the local processing requirements and data transmission overhead while still achieving high detection accuracy thanks to the spatial–spectral learning capacity of the CNN applied to the *GASF* representation. This design balances efficiency and performance, making it highly scalable for large CRNs.

## 5. Experimental Results

### 5.1. Real-Time Controlled Scenario

**Figure 8:**
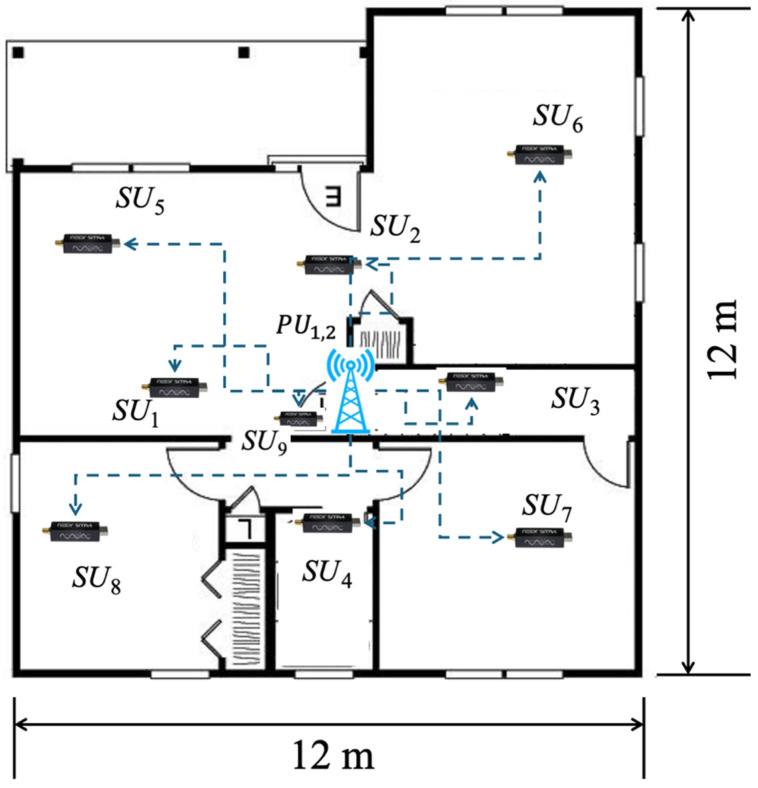
Real test environment (Adapted from Ref. [28]).

This section describes in detail the physical environment in which the proposed methodology, for evaluating PU behavior in a real wireless communication scenario, was implemented. In this scenario, illustrated in Figure 8, two PUs were strategically located in the center of the studied area, while nine SUs were distributed around them, covering different geographic areas of influence. Each SU was configured to detect the spectral activity of the PUs within its coverage range, reporting this information to both a shared database and a central entity in charge of consolidating the data. This infrastructure allowed for a more precise determination of the average number of PUs observed in the environment, the bandwidth used by these active PUs, their approximate location, and the coverage area of these transmissions.

It should be noted that this environment was previously used and characterized in [28], which presents a realistic wireless communication scenario. This proposed environment incorporates the presence of walls, doors, windows, and columns, which affect signal propagation, thus allowing for a more accurate and representative assessment of spectral behavior in real-world operating contexts. The spatial distribution of these elements can be seen in Figure 8, while Table 1 details the technical parameters used for configuring both the SUs and PUs.

These experimental settings, as discussed in [20], strongly affect the signal propagation in certain zones. Indeed, as a result of the configuration of the PUs and SUs in Figure 8, the presence of one of the PUs is not fully appreciated, due to its considerably low SNR. However, through the cooperation of other SUs in this proposed CRN, it is possible to mitigate this hidden terminal problem.

### 5.2. CNN Design

This section details the training process and architecture of the implemented CNN, aimed at interpreting and analyzing images generated from the average PSD signal frames.

#### 5.2.1. Training Stage

**Figure 9:**
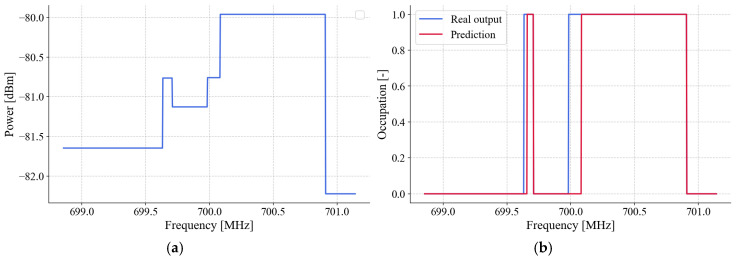
(**a**) Average PSD example. (**b**) CNN prediction and expected output (occupation).

The training environment considers a specific dataset comprising 2500 elements. Each element is made up of nine frames, each representing one of the nine secondary users described in the previous section. Each frame contains 732 average PSDs obtained from Algorithm 1 and represents the refresh time of the SDR devices, which, as mentioned before, corresponds to 100 ms. It is worth mentioning that 1024 points are originally calculated per frame; however, due to edge problems in the spectrum caused by the hardware of low-cost SDRs, it is then necessary to crop part of the spectral data, leaving only 732 useful points of the spectrum. Next, each set (element) is converted into nine images using the *GASF* technique. These images are grayscale with a resolution of 732 × 732 pixels. The main objective is to tune the network parameters to optimize the prediction of a binary sequence of 732 elements, representing the spectrum occupancy, as shown in Figure 9.

During the training stage of the neural network, the inputs are represented by the average power values, as mentioned above. For each input provided, the network generates a set of 732 binary outputs, where each bit corresponds to a specific point in the frequency domain (Figure 9). A value of ‘1’ at the output indicates the presence of a possible transmission, while a value of ‘0’ suggests the absence of a signal, being interpreted as noise. This approach allows the spectral occupancy to be accurately characterized, facilitating the identification of active regions within the analyzed frequency spectrum. For model training and validation, a total of 22,500 frames were used. This dataset was internally divided into two separate subsets: approximately 80% of the data (18,000 frames) was allocated for training the model, while the remaining 20% (4500 frames) was reserved for validation during the tuning phase. This careful split ensured that hyperparameter adjustments and model selection were performed using data not directly seen during training, thereby helping to prevent overfitting. Additionally, to provide an unbiased evaluation of the model’s generalization capacity, an entirely independent test set of 4500 frames was used. These test frames were not included at any point in the training or validation processes, guaranteeing a fair and realistic assessment of the model’s performance on unseen data.

**Figure 10:**
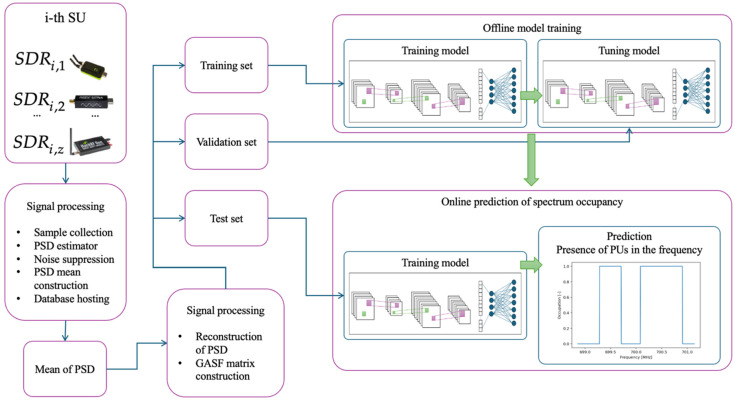
CNN training process.

The complete training process is illustrated in Figure 10. This processing involves real-time data acquisition within a real wireless communication environment that includes two PUs, as was explained in the preceding section.

#### 5.2.2. Architecture of the CNN

The CNN is designed to process the input images and predict the corresponding binary sequences. The CNN architecture is structured as follows:

Input layer: Accepts grayscale images with dimensions of 732 × 732 pixels.

Convolutional and pooling layers: Three convolutional layers with ReLU activation functions.

Each convolutional layer applies feature extraction to capture spatial patterns in the input images.

The model is trained using the Adam optimizer with an adjusted learning rate to improve convergence and stability during training.

### 5.3. System Performance Evaluation

System performance is assessed using training accuracy and validation accuracy metrics. These metrics are visualized through graphs at the end of each training cycle, allowing for continuous monitoring of the model’s learning process. This monitoring helps to identify potential issues such as overfitting (when the model memorizes the training data) or underfitting (when the model fails to capture underlying patterns). By analyzing these metrics, the training process can be adjusted to ensure better generalization to unseen data. This procedure enables robust model training, efficiently handling large datasets while allowing for the progressive optimization of the model based on the observed evaluation metrics. The real-time system performance was evaluated considering two fundamental metrics, the probability of success (*PS*) and the *F*1 score. To determine these metrics, four possible cases were considered:

- An analyzed window that corresponds to a PU transmission and that the SU classifies as a PU transmission is considered a true positive (*TP*) value.
- A frequency window that corresponds to a transmission of the PU that the SU classifies as noise is considered a false negative (*FN*) value.
- A window that corresponds to noise and that the SU classifies as a PU transmission, is considered a false positive (*FP*) value. A frequency window that corresponds to noise and that the SU classifies as noise, is considered a true negative (*TN*) value.

The *PS* is evaluated as follows:

(4) $$PS=\frac{TP+TN}{TP+FP+FN+TN},$$

and the *F*1 score as follows:

(5) $$F1=\frac{TP}{TP+0.5\left(FN+TN\right)}$$

being the result of counting the total of the correctly located frequency windows with respect to the total number of detected frequency windows.

**Figure 11:**
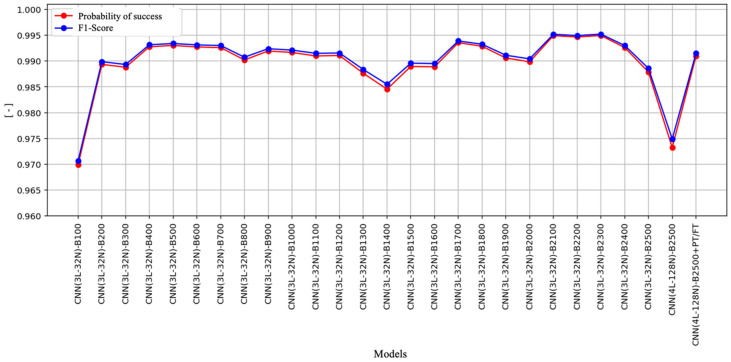
Results comparison from the evaluation of various CNN architectures using the *F*1 score and *PS* metrics.

The model used in this study is denoted as CNN(xL-yN)-Bz-[STAGES], as shown in Figure 11. This name indicates a CNN with x hidden layers, y neurons per layer, and a batch size of z*9 frames. Training can be performed in one or more stages: PT (pre- training), where the model is initially trained on a related dataset to learn general representations, and FT (fine-tuning), which consists of fine-tuning on the specific dataset, improving accuracy on the final task.

Figure 11 presents the results of the evaluation of various CNN architectures using these metrics. In this figure, we can observe that all architectures achieve a probability of success close to 1.0 (or 100%), which indicates that the models have an accurate and stable performance when classifying the samples. This behavior suggests that, regardless of the variations in the CNN configuration, the system achieves high accuracy in identifying the expected outputs. The graph on the right shows the *F*1 score obtained by each model, which also reaches values close to 1.0 in all configurations. Since the *F*1 score is a metric that combines precision (proportion of correct predictions) and exhaustiveness (ability to detect positive cases), these results suggest that the networks not only get their predictions right but also have a low incidence of errors by minimizing false positives and false negatives.

It is important to highlight the uniformity of the results across the different architectures evaluated. Despite variations in parameters such as the number of layers, filters, and training epochs, no significant differences are observed in the performance of the models. This stability indicates that the approach adopted is robust and reliable, which ensures consistent performance against different configurations. Furthermore, the homogeneous behavior of the metrics suggests that the CNNs evaluated do not present overfitting to the training data, since they maintain a high performance in both metrics. This aspect is essential to guarantee the generalization capacity of the model, that is, its ability to make accurate predictions on previously unseen data.

The results obtained with the current proposal, achieving an approximate accuracy of 99% in the localization of the PUs, show a performance comparable to that obtained with the previous methodology described in [28]. However, the approach presented here, which employs a convolutional neural network along with GAF images of the spectral signal, requires significantly less preprocessing. This reduction in preprocessing allows a substantial improvement in the refresh time required for SDR tuning, enabling a reduction of up to 50% in this parameter, that is, going from 100 ms to only 50 ms. To implement and evaluate the system, a Lenovo ThinkCentre desktop computer with an Intel Core i7 processor and 32 GB of RAM (mainframe) was used. During the testing phase, the average GAF matrix construction time was 1.21 ms, while the average neural network evaluation time was 32.88 ms over 4500 frames.

It is important to point out that the base methodology was quantitatively compared with several existing methods in previous publications by the authors [28,30,31], where superior detection performance was demonstrated. In this work, we focus on validating that the proposed real-time implementation maintains a detection accuracy and probability of success comparable with those reported in reference [28], where qualitative comparisons and detailed evaluation against other methods are already presented. Therefore, although this article does not replicate all comparative analyses, it emphasizes that the system performance remains constant when implemented in real-time scenarios using GAFs and a CNN. Furthermore, thanks to the introduced optimizations, the processing time has been reduced by half, now achieving a refresh for each secondary entity of only 50 ms, which we consider one of the main contributions for applications in dynamic environments.

## 6. Conclusions

This paper proposes the use of GAFs in conjunction with a CNN for spectrum occupancy detection in a real wireless communication environment. The approach consists of transforming signals into two-dimensional representations using the GAF method, which allows the CNN to extract relevant features to identify spectrum usage patterns.

Based on the analysis performed in a specific wireless communication environment, it is demonstrated that the CNN can accurately determine spectrum occupancy, facilitating the identification of free or used bands. This methodology is especially useful in cognitive radio systems, where it is essential to detect available frequencies in real time to optimize spectrum usage. Furthermore, the proposed approach can be adapted to different communication scenarios, improving efficiency and accuracy in spectrum management.

The combined use of GAFs and a CNN offers a robust alternative to traditional threshold-based methods, as it allows the detection of complex patterns and adaptation to changing environmental conditions. This approach not only improves the ability to identify weak signals or interference but also reduces the need for manual intervention. Furthermore, the methodology is scalable, allowing its implementation in systems with multiple receivers or in dynamic environments with high spectral variability. Finally, the results obtained from this technique are integrated into an automated monitoring platform, providing a more detailed and real-time analysis.

Regarding processing time, the transformation of the signals through GAF introduces an additional computational cost, but the optimized architecture of the CNN allows predictions to be made in reduced times. The experiments carried out indicate that the model is capable of processing large volumes of data in a time frame suitable for real-time detection applications (100 ms, and even this could be reduced by half), maintaining a balance between precision and efficiency. In addition, the inference time can be adjusted by optimizing the input parameters and using specialized hardware, which facilitates its implementation in embedded systems or low-latency platforms.

The results show that the convolutional neural network architectures evaluated have a high performance and a consistent generalization capacity. The high probability of success and the *F*1 score, close to 1.0, validate the effectiveness of the model for the classification task, ensuring its reliability in future applications and its ability to adapt to different configurations without compromising accuracy.

Finally, the system is sufficiently robust to handle multiple SUs. Indeed, under this scheme, if we want to extend the network to consider a different scenario (including more PUs and SUs), this would be entirely feasible, given that the network evaluates patterns resulting from the GAF associated with the frequency points (which correspond to the PSD) from each SU. In this way, it is possible to obtain a new scenario (more SUs and new locations) by simply retraining this neural network with the new GAFs obtained from the added SUs.

## Figures and Tables

**Table 1:** Principal settings for primary and secondary users (Adapted from Ref. [28]).

Label	Device	Fc Tx [MHz]	Fc Rx [MHz]	Bandwidth [MHz]	Location Coordinate (X,Y) [m]
PU_1_	Mini LimeSDR	699.5	-	0.5	(0, 0)
PU_2_	HackRF ONE	700.5	-	1	(0, 0)
SU_1_	RTL-SDR	-	700	2.4	(−1.5, 0)
SU_2_	RTL-SDR	-	700	2.4	(0, 1.5)
SU_3_	RTL-SDR	-	700	2.4	(1.5, 0)
SU_4_	RTL-SDR	-	700	2.4	(0, −1.5)
SU_5_	RTL-SDR	-	700	2.4	(−3, 2)
SU_6_	RTL-SDR	-	700	2.4	(3, 3.5)
SU_7_	RTL-SDR	-	700	2.4	(3, −2.5)
SU_8_	RTL-SDR	-	700	2.4	(−3, −2.5)
SU_9_	RTL-SDR	-	700	2.4	(0,0)

## Data Availability

Data are contained within the article.

## Abbreviations

The following abbreviations are used in this manuscript:

CNN	Convolutional neural network
CR	Cognitive radio
CRN	Cognitive radio network
DNN	Deep neural network
DSP	Digital signal processing
*FN*	False negative
*FP*	False positive
*GADF*	Gramian angular difference field
GAF	Gramian angular field
*GASF*	Gramian angular summation field
LSTM	Long short-term memory
MBSS	Multiband spectrum sensing
ML	Machine learning
MRA	Multiresolution analysis
*PS*	Probability of success
PSD	Power spectral density
PU	Primary user
REM	Radio environment map
RNN	Recurrent neural network
SDR	Software-defined radio
SGD	Stochastic Gradient Descent
SNR	Signal-to-noise ratio
SU	Secondary user
*TN*	True negative
*TP*	True positive
